# 

**Published:** 1903-04

**Authors:** 


### PERSONALS.

At the inauguration of Governor-elect Pennypacker, of Pennsylvania, the following well-known veterinarians were among those who took part in the ceremonies: Drs. M. Moriarty, Gettysburg; Jos. Johnson, West Grove; H. B. Cox, Philadelphia; E. E. Tower, Montrose; A. S. Shimer, Altoona; A. R. May, Boiling Springs; and J. H. Oyler and Wm. Hughes, of Harrisburg.

Dr. R. W. Braithwaite, of Champaign, Illinois, conducts a large veterinary hospital in connection with his practice.

Dr. Jas. B. Rayner, of West Chester, Pa., was seriously ill with La Grippe in the latter part of January.

Dr. W. F. Heyde, of St. Louis, Missouri, has built an addition to his hospital.

Dr. Howard Baker, of the Class of 1902 of the Veterinary Department of the University of Pennsylvania, located at Pittsfield, Mass., reports much encouragement from the people of that city.

Dr. J. R. Mahaffy, of Wilmington, Delaware, is fathering the bill looking to the registration of all veterinarians in Delaware, and the establishment of a State Board of Veterinary Medical Examiners.

Dr. H. B. McDowell, of Middletown, Delaware, takes a keen interest in the work of the Department of Agriculture of the "Blue Hen State."

Dr. C. J. Waidner, of Hellertown, Pa., fills the rôle of County Commissioner and Prison Inspector for Northampton County.

Dr. W. C. Holden, of Delphos, Ohio, conducts a veterinary hospital in connection with his veterinary practice.

Dr. Alex. Glass, of Philadelphia, Pa., spent ten days in bed in the latter part of January from an attack of influenza.

Dr. C. A. Raque, graduate of the American Veterinary College, and formerly located at West Nyack, N. Y., is now an attaché of the Bureau of Animal Industry and located at Chicago, Ill.

Dr. David Martin, of McKeesport, Pa., is the proprietor of the McKeesport Veterinary Infirmary.

Dr. E. D. Hudson, of Fairfield, Pa., is also proprietor of the Mansion House of that city.

Dr. E. R. Hogg, of Wilkesbarre, was confined to his bed for ten days with the "grippe" the latter part of February.

Dr. J. W. Moore, of Waynesburg, Pa., takes a great interest in the subject of "good roads."

